# MiR-625-3p promotes cell migration and invasion via inhibition of SCAI in colorectal carcinoma cells

**DOI:** 10.18632/oncotarget.4738

**Published:** 2015-07-28

**Authors:** Hailun Zheng, Renqiang Ma, Qizhi Wang, Pei Zhang, Dapeng Li, Qiangwu Wang, Jianchao Wang, Huabin Li, Hao Liu, Zhiwei Wang

**Affiliations:** ^1^ Department of Gastroenterology, The First Affiliated Hospital of Bengbu Medical College, Bengbu, Anhui, China; ^2^ Cancer Center, ENT Hospital, The First Affiliated Hospital of Sun Yat-sen University, Guangzhou, China; ^3^ Faculty of Pharmacy, Bengbu Medical College, Biochemical Drugs Engineering and Technological Research Center of Anhui Province, Bengbu, Anhui, China; ^4^ The Cyrus Tang Hematology Center and Collaborative Innovation Center of Hematology, Jiangsu Institute of Hematology, The First Affiliated Hospital, Soochow University, Suzhou, China

**Keywords:** miR-625-3p, SCAI, invasion, migration, colorectal carcinoma

## Abstract

MicroRNAs (miRNAs) play a critical role in controlling tumor invasion and metastasis via regulating the expression of a variety of targets, which act as oncogenes or tumor suppressor genes. Abnormally expressed miR-625-3p has been observed in several types of human cancers. However, the molecular mechanisms of miR-625-3p-mediated tumorigenesis are largely elusive. Therefore, the aim of this study was to evaluate the biological function and molecular insight on miR-625-3p-induced oncogenesis in colorectal carcinoma (CRC). The effects of miR-625-3p in cell migration and invasion were analyzed by wound healing assay and transwell assay, respectively. In addition, the expression of miR-625-3p and its targets was detected in five human CRC cell lines. In the present study, we found that overexpression of miR-625-3p promoted migration and invasion in SW480 cells, whereas downregulation of miR-625-3p inhibited cell motility in SW620 cells. More importantly, we observed potential binding sites for miR-625-3p in the 3′-untranslated region of suppressor of cancer cell invasion (SCAI). Notably, we identified that overexpression of miR-625-3p inhibited the expression of SCAI, while depletion of miR-625-3p increased SCAI level, suggesting that SCAI could be a target of miR-625-3p. Additionally, we revealed that miR-625-3p exerts its oncogenic functions through regulation of SCAI/E-cadherin/MMP-9 pathways. Our findings indicate the pivotal role of miR-625-3p in invasion that warrants further exploration whether targeting miR-625-3p could be a promising approach for the treatment of CRC.

## INTRODUCTION

Colorectal cancer (CRC) is the third most common tumor and the primary cause of cancer-related death worldwide [1]. The main causes of death in CRC patients are tumor cell invasion and metastasis, which is a complex process that involves changes in the extracellular matrix, leading to translocation from the primary tumor to a distant organ with maintenance of growth at a distant site [2]. Although considerable progress has been made over the past decades, the molecular mechanisms underlying tumor invasion and metastasis remain largely unclear. Therefore, it is pivotal to explore the mechanisms of tumor metastasis and to develop the novel strategy for CRC treatment.

Recent accumulating evidence suggests that microRNAs (miRNAs) play a critical role in the regulation of multiple cellular processes during cancer development and progression, including tumor invasion and metastasis [3, 4]. It has been well documented that miRNAs are short endogenous non-coding RNAs that are involved in cellular functions, including proliferation, apoptosis, differentiation and metabolism [5]. Clearly, miRNAs regulate gene expression through binding to complementary sequences, preferentially the 3′-untranslated region (3′-UTR) of target messenger RNA (mRNA), to inhibit translation or promote RNA degradation and even to regulate mRNA transcription [6–8]. A series of studies have shown that deregulation of miRNAs is a common event in tumorigenesis, and correlates with disease stage, metastasis and survival in a variety of human malignancies including CRC [9]. Specifically, some miRNAs acting as tumor suppressors are downregulated in CRC, whereas other miRNAs as tumor promoters are over-expressed in cancer tissue compared with normal tissue [10–12]. Thus, investigating tumor-specific miRNAs and their targets is critical to understand their role in tumorigenesis and to identify new molecular markers for the diagnosis, prognosis, and treatment of CRC. These findings further indicate that targeting miRNAs could be useful for treating human cancers with aberrant miRNAs expression [13, 14].

Some studies have revealed that miRNAs govern the tumor migration, invasion, and metastasis in CRC [15–17]. For example, miR-182 promotes cell growth and invasion through targeting forkhead box F2 transcription factor in colorectal cancer [17]. Chai et al. found that miR-455 inhibited proliferation and invasion of colorectal cancer through inhibiting RAF (rapidly accelerated fibrosarcoma) proto-oncogene serine/threonine-protein kinase [15]. Lu et al. reported that miR-185 suppressed cell growth and invasion via targeting the hypoxia-inducible factor-2α pathway *in vitro* and *in vivo* in colorectal cancer [16]. Similarly, hsa-miR-140-5p inhibited colorectal cancer stem cell survival and invasive potential via suppression of Smad2 (mothers against decapentaplegic homolog 2) and autophagy [18]. Moreover, hsa-miR-574-5p was found to negatively regulate MACC-1 (metastasis associated in colon cancer 1) expression to inhibit colorectal cancer liver metastasis [19]. Furthermore, miR-132 inhibited colorectal cancer invasion and metastasis through targeting ZEB2 (zinc finger E-box binding homeobox 2) [20]. In addition, miR-128, miR-134, and miR-330 targeted the MMP-3 (matrix metalloproteinase), MMP-10, and MMP-13, respectively, in a mouse model of chemically induced colitis-associated cancer [21]. Emerging evidence has also supported that multiple miRNAs such as miR-200c [22, 23], miR-153 [24], miR-126 [25, 26], miR-19a [27], miR-32 [28], govern the cell invasion and metastasis in CRC through targeting their specific targets. Taken together, miRNAs are critically involved in tumor invasion and metastasis in CRC.

A recent series of studies demonstrated that miR-625-3p, one member of miR-625 family, contributed to tumor development, progression and metastasis in malignant mesothelioma and CRC [29, 30]. Importantly, increased circulating miR-625-3p could be a potential biomarker for patients with malignant pleural mesothelioma (MM) [30]. Consistent with this, high expression of miR-625-3p has also been associated with poor response to first-line oxaliplatin-based treatment for metastatic colorectal cancer [29]. However, the mechanism and function of miR-625-3p in CRC have not been fully determined. Therefore, in the current study, we investigated whether miR-625-3p plays an important role in controlling cancer cell migration and invasion in CRC by overexpression or depletion of miR-625-3p expression in CRC cells. We further explored whether miR-625-3p exerts its oncogenic function via inhibiting its target SCAI (suppressor of cancer cell invasion). We also determine whether E-cadherin/MMP-9 pathway is involved in miR-625-3p-mediated tumorigenesis in CRC.

## RESULTS

### miR-625-3p expression is associated with invasive activity in CRC cell lines

**Figure 1:**
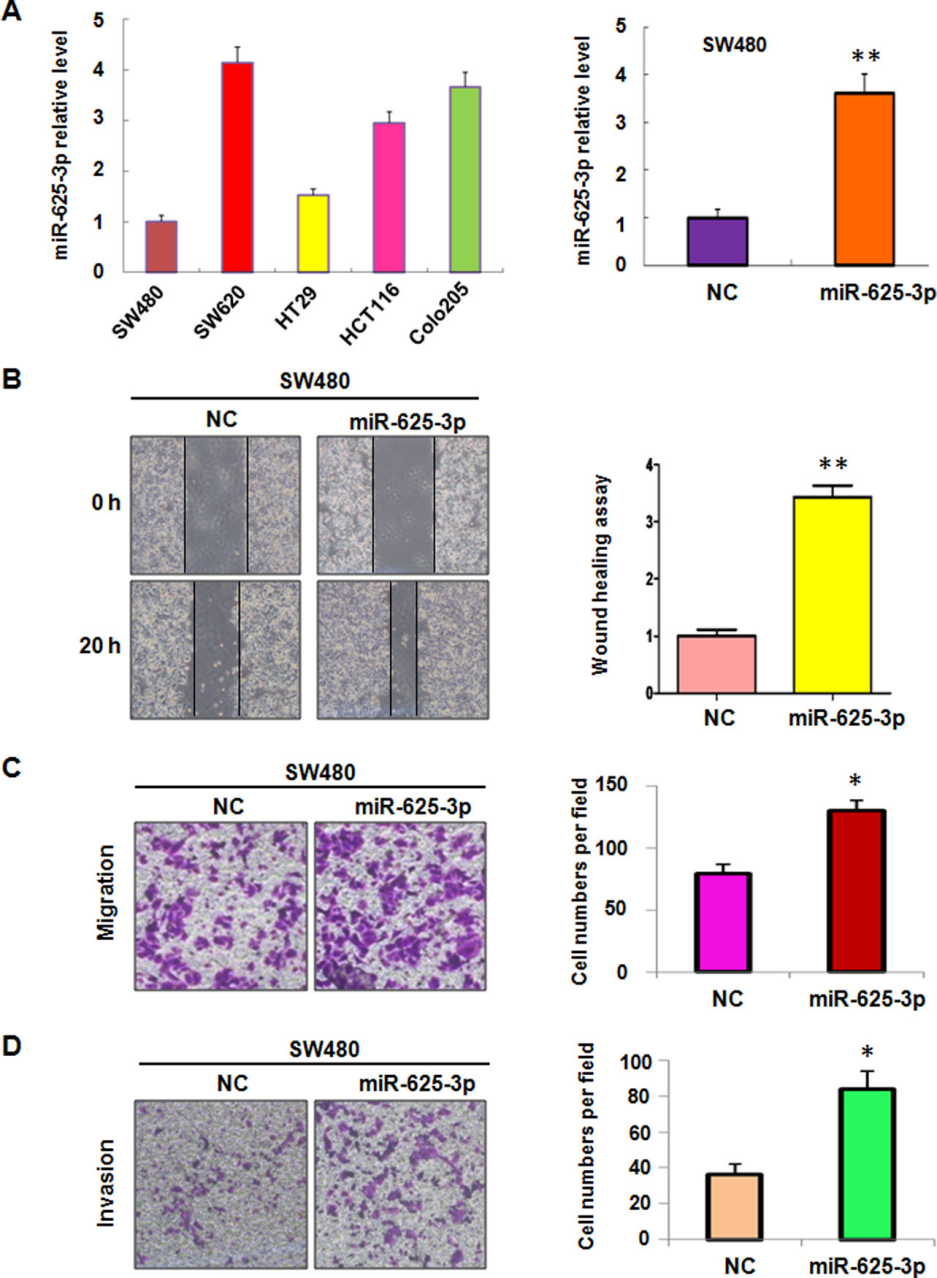
Over-expression of miR-625-3p promoted cell migration and invasion in SW480 cells NC: negative control; miR-625-3p: miR-625-3p mimic. *P < 0.05; **P < 0.01 vs control. **A.** miR-625-3p level was measured by real-time RT-PCR in five CRC cell lines (left panel) and SW480 cells with miR-625-3p mimic treatment (right panel). **B.** Left panel, the cell motility was detected using wound healing assay in SW480 cells transfected with miR-625-3p. Right panel, Quantitative results are illustrated for left panels. **C-D.** Left panel, the cell migration and invasion were detected by uncoated (C) and coated (D) Transwell chambers assay. Right panel, Quantitative results are illustrated for left panel.

To better understand the association between miR-625-3p and cell invasion, the baseline expression of miR-625-3p was measured in a panel of human colorectal cancer cell lines that included SW480, SW620, HT29, HCT116, and Colo205. The results showed that miR-625-3p was frequently but differentially expressed in different human colorectal cancer cell lines (Figure 1A). Specifically, we observed the higher expression of miR-625-3p in SW620, HCT116, and Colo205 cells (Figure 1A). It has been known that SW480 and HT29 cells are moderate differentiation, whereas SW620, HCT116 and Colo205 cells are poor differentiation with high invasive activity [31]. Our results indicated that miR-625-3p could promote cell invasion in CRC cells. Studies have demonstrated that SW620 and Colo205 cells exhibited metastasis feature [31]. Consistent with this notion, we found that miR-625-3p expression was higher in SW620 and Colo205 cells compared with that of other three cell lines (Figure 1A). These data suggest that miR-625-3p could be involved in regulation of invasion in CRC cells.

### Over-expression of miR-625-3p promoted cell migration and invasion

SW480 cells were chosen for further exploring the function of miR-625-3p through overexpressing strategy by its mimics due to that SW480 cells showed lowest expression of miR-625-3p. The level of miR-625-3p was detected using real-time RT-PCR in miR-625-3p mimic transfected cells. Our results showed that miR-625-3p was markedly increased by its mimics treatment (Figure 1A). Next, we measured the cell motility and invasion capacities in SW480 cells transfected with miR-625-3p mimics. The wound healing assay showed that miR-625-3p promoted the cell motility in SW480 cells (Figure 1B). Consistent with this, overexpression of miR-625-3p enhanced the migration and invasion in SW480 cells (Figure 1C, 1D). Overexpression of miR-625-3p did not increase the cell proliferation at 24 hours (data not shown), suggesting that miR-625-3p governed the cell invasive activity not due to regulation of cell proliferation. Taken together, our findings demonstrated that miR-625-3p is involved in promoting cell migration and invasion in CRC cells.

### Inhibition of miR-625-3p reduced cell motility and invasion

**Figure 2:**
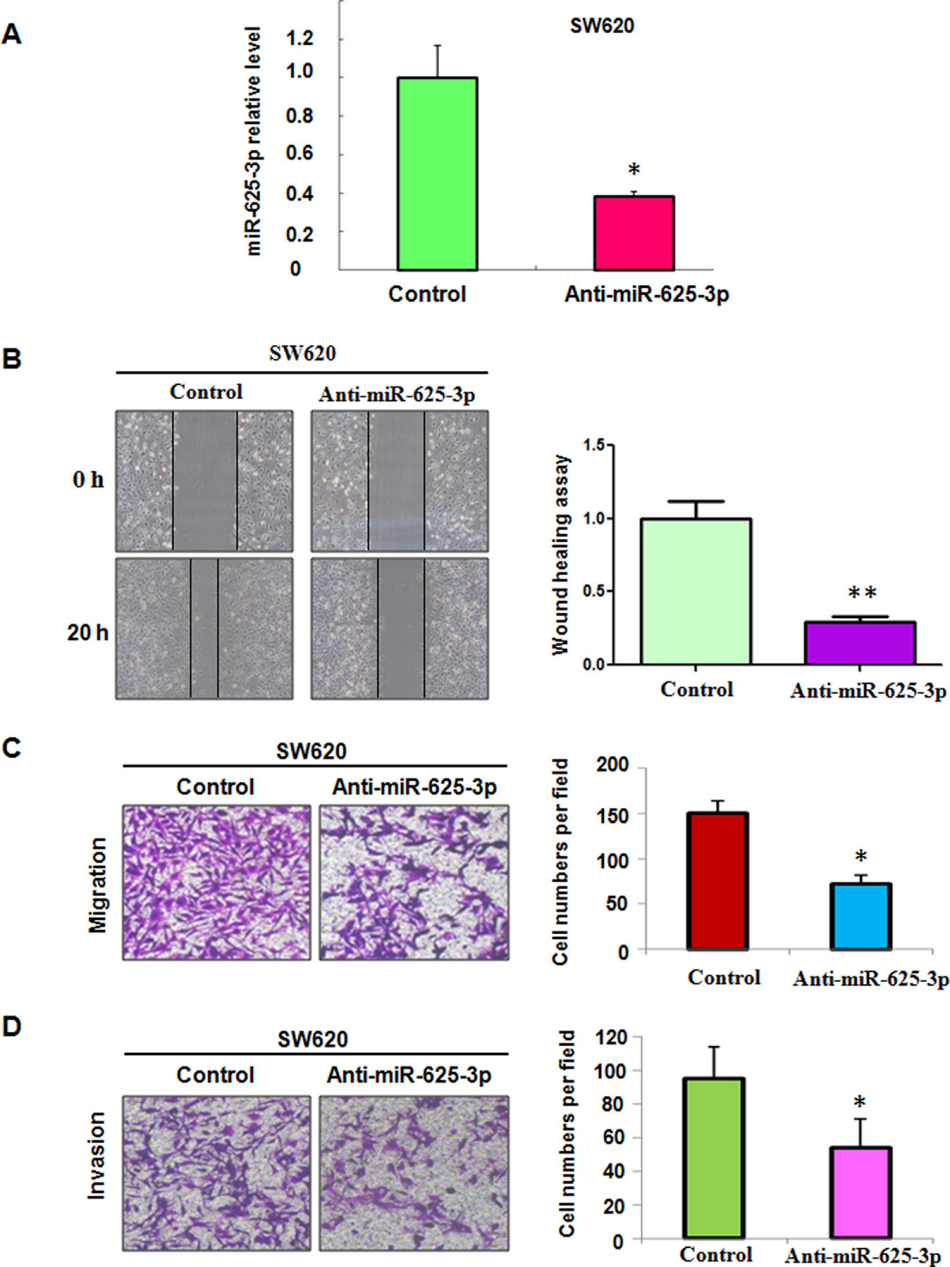
Down-regulation of miR-625-3p inhibited cell migration and invasion in SW620 cells Anti-miR-625-3p: miR-625-3p inhibitor. *P < 0.05; **P < 0.01 vs control. Control: anti-miR-control. **A.** miR-625-3p level was determined by real-time RT-PCR in SW620 cells with miR-625-3p inhibitor treatment. **B.** Left panel, the cell motility was measured using wound healing assay in SW620 cells treated with miR-625-3p inhibitor. Right panel, Quantitative results are illustrated for left panels. **C-D.** Left panel, the cell migration and invasion were measured using uncoated (C) and coated (D) Transwell chambers assay. Right panel, Quantitative results are illustrated for left panel.

To further validate the role of miR-625-3p in CRC cells, SW620 cells with highest expression of miR-625-3p were used. As illustrated in Figure 2A, our real-time RT-PCR confirmed that miR-625-3p inhibitors significantly decreased the expression of miR-625-3p in SW620 cells. Moreover, downregulation of miR-625-3p retarded the cell motility (Figure 2B, 2C). Moreover, SW620 cells treated with miR-625-3p inhibitor showed a low level of penetration through the matrigel-coated membrance compared with the control cells (Figure 2D). Altogether, miR-625-3p plays a key role in governing cell migration and invasion.

### SCAI is a potential downstream target of miR-625-3p

**Figure 3:**
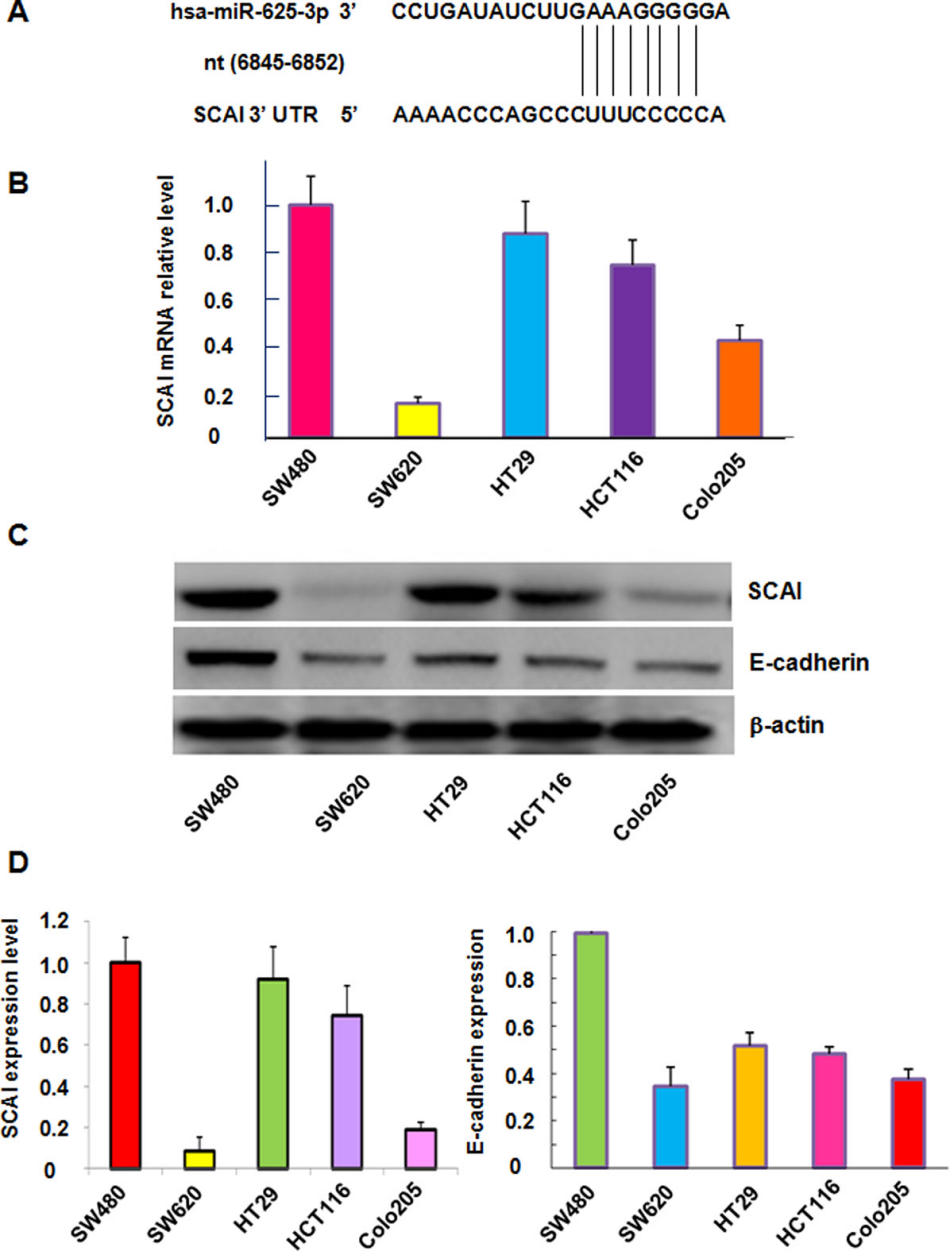
The expression of miR-625-3p, SCAI and E-cadherin was correlated in CRC cells **A.** SCAI sequence analysis indicates that it harbors potential miR-625-3p target sites, which are nt 6845–6852 sequences of the SCAI 3′ UTR. **B.** SCAI mRNA level was detected by real-time RT-PCR in CRC cell lines. **C.** Western blotting analysis was performed to detect the SCAI protein level in CRC cell lines. **D.** Quantitative results are illustrated for panel C.

To further understand the molecular mechanism of miR-625-3p-mediated invasion in CRC cells, we sought to identify the target of miR-625-3p. SCAI sequence analysis revealed that it harbors potential miR-625-3p target sites, which are nt 6845–6852 sequences of the SCAI 3′ UTR (Figure 3A). To explore the correlation between miR-625-3p and SCAI, we measured the SCAI mRNA levels in five CRC cell lines. Our results from RT-PCR demonstrated that SCAI expression at mRNA level was dramatically reduced in metastatic CRC cells (SW620 and Colo205) compared with non-metastatic cell lines including SW480, HCT116 and HT29 (Figure 3B). Our Western blotting analysis further confirmed the SCAI protein levels were also down-regulated in metastatic CRC cells compared with non-metastatic cells (Figure 3C, 3D). Next, we measured the expression of E-cadherin in five CRC cell lines because SCAI has been reported to regulate E-cadherin [32]. As expected, like SCAI, the expression of E-cadherin was decreased in metastatic CRC cells (Figure 3C, 3D). Our findings demonstrated that miR-625-3p could promote cell migration and invasion through inhibition of SCAI and its target E-cadherin.

### Over-expression of miR-625-3p inhibited SCAI expression in SW480

**Figure 4:**
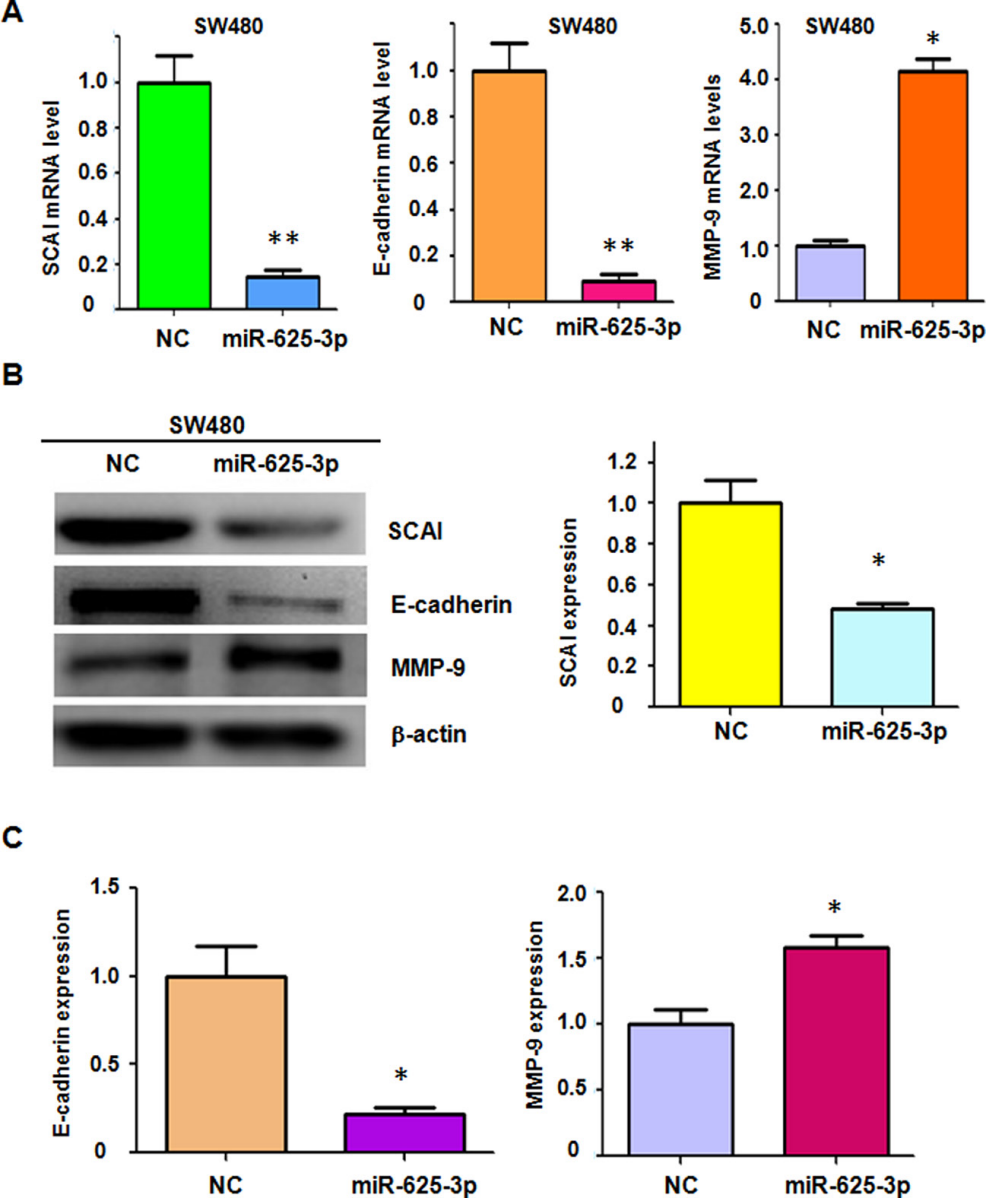
Over-expression of miR-625-3p inhibited SCAI expression in SW480 NC: negative control; miR-625-3p: miR-625-3p mimic. *P < 0.05; **P < 0.01 vs control. **A.** Real-time RT-PCR was conducted to detect the expression of SCAI, E-cadherin, and MMP-9 at the mRNA levels in SW480 cells transfected with miR-625-3p mimic. **B.** Western blotting analysis was performed to detect the expression of SCAI, E-cadherin, and MMP-9 in SW480 cells transfected with miR-625-3p mimic (left panel). Quantitative result of SCAI expression is presented (right panel). **C.** Quantitative results are illustrated for panel B.

To address whether SCAI is a target of miR-625-3p, we detected the expression of SCAI in SW480 cells transfected with miR-625-3p mimics by real-time RT-PCR and Western blotting analysis. We found that overexpression of miR-625-3p significantly reduced the SCAI expression at mRNA and protein levels in SW480 cells (Figure 4A, 4B). Moreover, E-cadherin mRNA and protein levels were also decreased in SW480 cells transfected with miR-625-3p mimics (Figure 4A–4C). Several studies have revealed that E-cadherin could down-regulate MMP-9 expression in human tumor cells [33, 34]. Therefore, we also investigated the expression of MMP-9 in miR-625-3p-transfected SW480 cells. We observed that MMP-9 expression was upregulated in SW480 cells after miR-625-3p mimics treatment (Figure 4B, 4C). These results suggested that miR-625-3p regulated migration and invasion at least in part through SCAI/E-cadherin/MMP-9 pathway.

### Down-regulation of miR-625-3p increased SCAI expression

**Figure 5:**
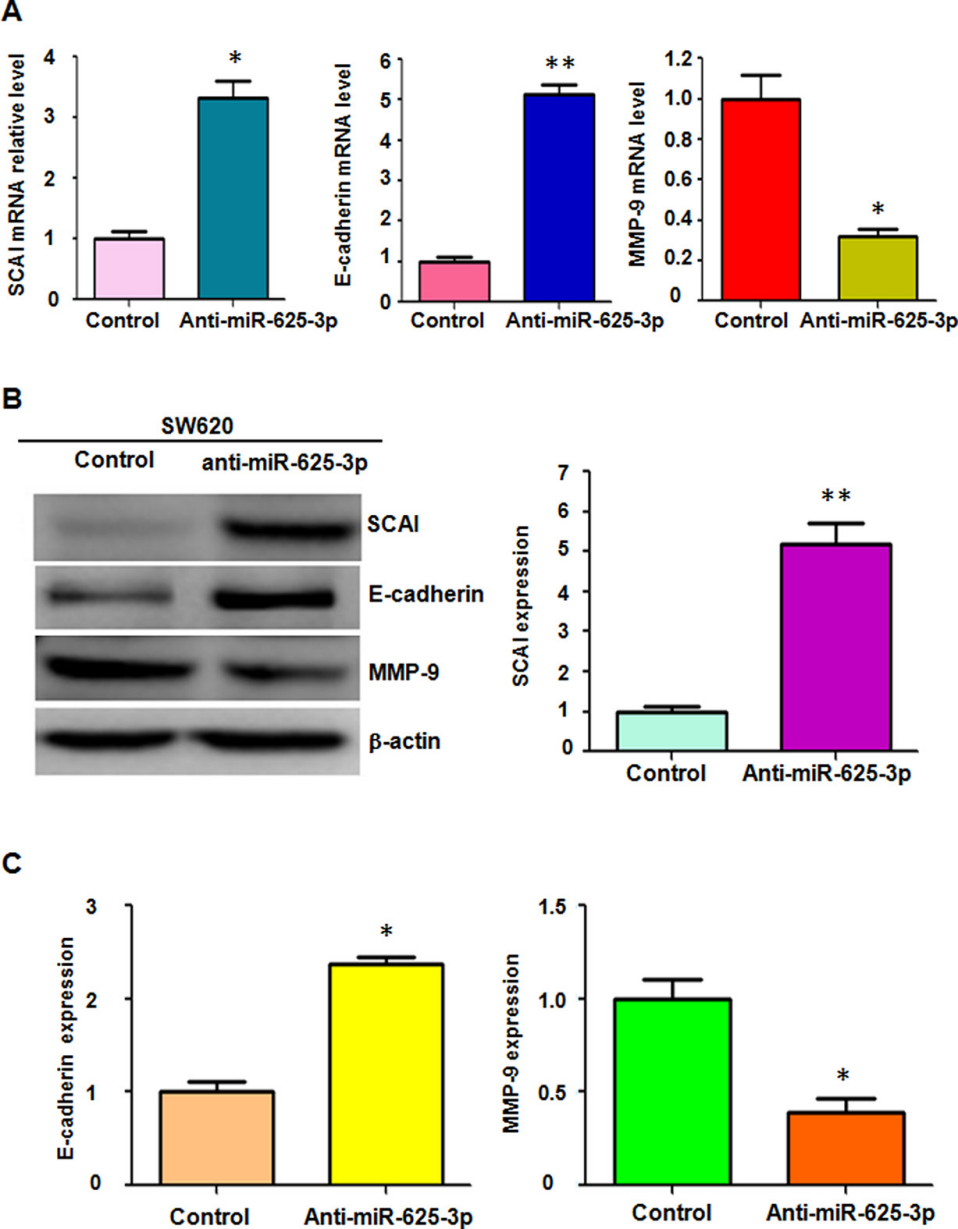
Down-regulation of miR-625-3p increased SCAI expression Anti-miR-625-3p: miR-625-3p inhibitor. *P < 0.05; **P < 0.01 vs control. **A.** Real-time RT-PCR was performed to detect the expression of SCAI, MMP-9, and E-cadherin at the mRNA levels in SW620 treated with miR-625-3p inhibitor. **B.** Western blotting analysis was used to measure the expression of SCAI, E-cadherin, and MMP-9 in SW620 cells with miR-625-3p inhibitor treatment (left panel). Quantitative result of SCAI expression is presented (right panel). **C.** Quantitative results are illustrated for panel B.

To further verify whether miR-625-3p regulates SCAI/E-cadherin/MMP-9 pathway in CRC cells, we used Western blotting analysis and RT-PCR to measure SCAI, E-cadherin, and MMP-9 levels in SW620 cells after miR-625-3p inhibitor treatment. We observed that depletion of miR-625-3p upregulated SCAI and E-cadherin expressions, but down-regulated MMP-9 level in SW620 cells (Figure 5A-5C). Due to that E-cadherin and MMP-9 play a critical role in controlling migration and invasion, miR-625-3p could inhibit SCAI and subsequently govern E-cadherin and MMP-9 expression, leading to enhanced invasion in CRC cells.

## DISCUSSION

In the current study, we explored the molecular mechanism of miR-625-3p-meidated tumorigenesis in CRC cells. Our data demonstrated that miR-625-3p level is associated with invasive activity in CRC cell lines. Moreover, our findings revealed that miR-625-3p enhanced cell migration and invasion in CRC cells. Up-regulation of miR-625-3p promoted cell motility, while down-regulation of miR-625-3p inhibited cell invasive activity in CRC cells. Strikingly, we identified SCAI as a potential target of miR-625-3p. Notably, we have evidenced that miR-625-3p exerts its oncogenic function in enhancing cell invasion via regulation of SCAI/E-cadherin/MMP-9 pathways. Our study thus offers a new strategy to treat CRC through inhibiting miR-625-3p level.

Accumulating evidence has demonstrated that miR-625-3p is highly expressed in human cancers [29, 30]. For instance, miR-625-3p was present in significantly higher concentration in plasma/serum from malignant mesothelioma patients, which can discriminate between patients and control group [30]. Consistently, upregulation of miR-625-3p was observed in tumor specimens from malignant mesothelioma patients [30]. This study indicated that miR-625-3p could be a novel diagnostic marker for malignant mesothelioma. Another study showed that miR-625-3p was up-regulated in oxaliplatin resistant CRC cell lines, suggesting that strong expression of miR-625-3p is positively correlated with a poor response to first-line oxaliplatin treatment of metastatic CRC [29]. Interestingly, miR-625-3p was not associated with recurrence of stage II or III disease [29], indicating that further investigation is required to determine the role of miR-625-3p in CRC. To this end, our present study identified that higher expression of miR-625-3p was found in CRC cell lines with poor differentiation, high invasive activity and metastasis characteristics. Consistent with this, our results support that miR-625-3p controls cell migration and invasion in CRC cells.

Emerging evidence has indicated that SCAI inhibited migration and invasion in human cancers, and SCAI is downregulated in a variety of human tumors [35–37]. An elegant study defined that SCAI suppressed cancer cell invasion through the transcriptional control of β1-integrin [37, 38]. Chen et al. found that downregulation of SCAI promoted cell invasion and stem cell like phenotype through activation of Wnt/beta-catenin signaling in glioma [36]. Further study demonstrated that SCAI interacts with the tumor suppressing SWI/SNF (SWItch/Sucrose nonfermentable) chromatin remodeling complex to regulate gene expression and promote invasive capacities of human cancer cells [39]. Moreover, SCAI was identified as a novel transcriptional cofactor that regulates EMT [32]. Li et al. reported that miR-1290 promoted cell proliferation and metastasis through inhibiting SCAI in esophageal squamous cell carcinoma [35]. Here, our data suggest that miR-625-3p inhibited SCAI and led to increased migration and invasion in CRC cells. We believe that multiple other miRNAs could be involved in regulating SCAI and further exploration is needed.

It has been reported that SCAI can rescue TGF-β1-induced E-cadherin down-regulation [32], indicating that SCAI governs E-cadherin expression. It has been known that Cadherins play a critical role in cell adhesion and maintenance of normal tissue structures [40]. The disturbance of cadherin-dependent cell interactions is one of reasons that leads to cancer cell invasiveness and metastases [41]. The dysregulated E-cadherin expression could be a key indicator of malignant cancer development and progression. As E-cadherin is an important factor for cell invasion, miR-625-3p promoted cell invasion partly via modulating SCAI/E-cadherin. Matrix metalloproteinases are a family of extracellular matrix (ECM) degrading enzymes, which provide a permissive microenvironment for tumor invasion and metastasis [42]. It has been demonstrated that MMP-9 is involved in the proteolytic cascade-leading ECM cleavage that occurs during CRC metastasis [43, 44]. Moreover, MMP-9 regulated vascular endothelial growth factor-mediated neoangiogenesis of colorectal cancer, and MMP-mediated endoglin mobilisation plays a key role in the regulation of the angiogenic potential of endothelial cells in colorectal cancer [45]. Importantly, E-cadherin has been reported to inhibit MMP-9 expression [33, 34]. Therefore, miR-625-3p-mediated CRC cell migration and invasion may be partly through regulating SCAI/E-cadherin/MMP-9 pathway in CRC.

**Figure 6:**
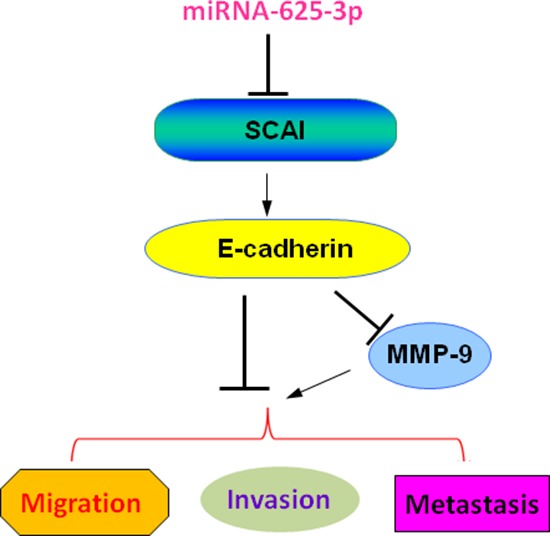
A schematic illustration of the signaling network showing how miR-625-3p promotes cell migration and invasion MiR-625-3p inhibited SCAI expression and subsequently suppressed E-cadherin and upregulated MMP-9 expression, leading to enhanced cell migration, invasion and metastasis in CRC.

To this end, we found for the first time that miR-625-3p markedly inhibited SCAI expression and subsequently suppressed E-cadherin and upregulated MMP-9 expression, leading to enhanced cell invasion in CRC (Figure 6). Without a doubt, further in-depth investigation is necessary to fully elucidate the molecular insight on miR-625-3p-triggerd cell invasion in human CRC cells. In summary, our findings reveal the critical role of miR-625-3p in cell invasion and indicate that downregulation of miR-625-3p may be a promising novel approach for the treatment of CRC.

## MATERIALS AND METHODS

### Cell culture

Human colorectal carcinoma cell lines SW480, SW620, HT29, HCT116, and Colo205 were cultured in Dulbecco’s modified Eagle medium (DMEM) supplemented with 10% fetal bovine serum (FBS) and 100 U of penicillin-streptomycin in a humidified atmosphere of 95% air and 5% CO_2_ incubator at 37°C. SW480 and SW620 cell lines with different invasive and migratory abilities were collected from the same patient with colorectal carcinoma. SW480 cells were obtained from the primary site, whereas SW620 cells were from a lymph node metastatic site [31].

### Reagents and antibodies

DMEM, fetal bovine serum (FBS), and phosphate buffered saline (PBS) were purchased from Gibco (Grand Island, NY). Primary antibodies for SCAI, MMP-9, E-cadherin, and β-actin were obtained from Santa Cruz Biotechnology Co., Ltd. (Santa Cruz, CA, U.S.).

### miR-625–3p transfection

SW480 cells were transfected with miR-625-3p mimics (Invitrogen, Shanghai, China) or a miR-625-3p mimics control (negative control, NC) (Invitrogen) using the Lipofectamine 2000 reagent (Invitrogen) following the manufacturer’s protocol. SW620 cells were transfected with miR-625-3p inhibitor (anti-miR-625-3p, Invitrogen) or inhibitor NC (anti-miR-control, Invitrogen) using the Lipofectamine 2000 reagent. Forty-eight hours after transfection, cells were collected for further assays as described before [46].

### Wound healing assay

The capability of cell migration was examined by wound healing assay. Briefly, SW480 or SW620 cells were seeded on 6-well plates at 5 × 10^5^ cells/well. Cells were transfected with the miR-625-3p mimic or inhibitor when they reached 90% confluence. Following starvation for 24 h, artificial wounds were created by insertion of a sterile 200 μL pipette tip into cells, and then washed 3 times with PBS to remove floating cells and debris. Migration at the wound site was observed and representative images captured under an inverted microscope at 0 h and 20 h to calculate healing percentage as described before [47].

### Cell migration and invasion assay

Cell migration and invasion were evaluated using 24-well uncoated and coated transwell cell culture chambers, respectively, as previously described [46]. Briefly, 1 × 10^5^ transfected cells were re-suspended in 200 μl serum-free medium and placed in the upper chamber with the lower chamber containing 600 μl DMEM and 10% FBS. After 24 hours, cells on the lower chamber were fixed with 4% paraformaldehyde in PBS buffer and stained with 0.1% crystal violet. The migrated cells were counted and photographed at 200× magnification in 5 different fields per filter.

### RNA isolation and quantitative real-time RT-PCR

Total RNA was extracted from the cell lines SW480, SW620, HT29, HCT116, and Colo205, using TRIzol reagent (Invitrogen, Carlsbad, CA, USA) according to the manufacturer’s instructions. The primers for RT–PCR were designed and synthesized by the Takara Co., Ltd (Kyoto, Japan.). The RT-PCR was performed according to miR-625-3p qPCR Quantitation Kit (Takara, Kyoto, Japan). All RT reactions were performed by the iScript cDNA synthesis kit (Takara, Kyoto, Japan) in an ABI StepOne™ Real-Time PCR System (Applied Biosystems, Foster City, CA, U.S.) with U6 as internal control. Aliquots (1 μg) of RNA were reverse transcribed to cDNA (20 μL) with oligo (dT) and M-MuLV reverse transcriptase (Fermentas, Glen Burnie, MD, U.S.) in accordance with the manufacturer’s instructions. One-fifth of the cDNA was used as a template for PCR using the SYBR Green PCR kit (Takara, Kyoto, Japan) in an ABI StepOne™ Real-Time PCR System (Applied Biosystems, Foster City, CA, U.S.). The housekeeping gene, glyceraldehyde-3-phosphate dehydrogenase (GAPDH), served as an internal control for each experiment. The primer sequences for target genes were as follows: SCAI (forward, 5′-ACC CCT GTT CAT CGT TGT G-3′; reverse, 5′-CGA GTG GCT GTC CAA ACA A-3′; E-cadherin (forward, 5′-TCG ACA CCC GAT TCA AAG TGG-3′; reverse, 5′-TTC CAG AAA CGG AGG CCT GAT-3′); MMP-9 (forward, 5′-CGG AGT GAG TTG AAC CAG-3′; reverse, 5′-GTC CCA GTG GGG ATT TAC-3′); GAPDH (forward, 5′-ACG GGA AGC TCA CTG GCA TGG-3′; reverse, 5′-GGT CCA CCA CCC TGT TGC TGT A-3′). Cycling conditions were as follows: an initial 10 min of pre-denaturation at 95°C, followed by 40 cycles of 95°C for 10 s, 60°C for 20 s, and 72°C for 15 s. The specificity of the amplification products was confirmed by melting curve analysis. The mRNA was quantified using the 2^−ΔΔCt^ method as described before [46].

### Western blotting analysis

Cell extracts were prepared using a cell lysis reagent (Sigma, St. Louis, MO, USA) according to the manual and protein concentrations detected using a bicinchoninic acid (BCA) assay. Equal amounts of total protein were separated by 10% SDS-PAGE and transferred to polyvinylidene fluoride (PVDF) membranes. After blocking in 5% non-fat milk, membranes were incubated with primary antibodies overnight at 4°C followed by incubation in secondary antibodies for 2 h at room temperature on a shaker. The bands were visualized using Western Lightning ECL Pro with horseradish peroxidase (HRP) and then exposed to medical X-ray films for seconds or minutes. β-actin was used as a loading control [47].

### Statistical analysis

All assays were performed in triplicate and data are expressed as mean ± S.D. Differences between groups were analyzed using the two-tailed Student’s *t*-test for two groups. *p* value < 0.05 was considered significant.
